# Vertical ground reaction force marker for Parkinson’s disease

**DOI:** 10.1371/journal.pone.0175951

**Published:** 2017-05-11

**Authors:** Md Nafiul Alam, Amanmeet Garg, Tamanna Tabassum Khan Munia, Reza Fazel-Rezai, Kouhyar Tavakolian

**Affiliations:** 1 Department of Electrical Engineering, University of North Dakota, Grand Forks, North Dakota, United States of America; 2 School of Engineering Science, Simon Fraser University, Burnaby, British Columbia, Canada; Northwestern University, UNITED STATES

## Abstract

Parkinson’s disease (PD) patients regularly exhibit abnormal gait patterns. Automated differentiation of abnormal gait from normal gait can serve as a potential tool for early diagnosis as well as monitoring the effect of PD treatment. The aim of current study is to differentiate PD patients from healthy controls, on the basis of features derived from plantar vertical ground reaction force (VGRF) data during walking at normal pace. The current work presents a comprehensive study highlighting the efficacy of different machine learning classifiers towards devising an accurate prediction system. Selection of meaningful feature based on sequential forward feature selection, the swing time, stride time variability, and center of pressure features facilitated successful classification of control and PD gaits. Support Vector Machine (SVM), K-nearest neighbor (KNN), random forest, and decision trees classifiers were used to build the prediction model. We found that SVM with cubic kernel outperformed other classifiers with an accuracy of 93.6%, the sensitivity of 93.1%, and specificity of 94.1%. In comparison to other studies, utilizing same dataset, our designed prediction system improved the classification performance by approximately 10%. The results of the current study underscore the ability of the VGRF data obtained non-invasively from wearable devices, in combination with a SVM classifier trained on meticulously selected features, as a tool for diagnosis of PD and monitoring effectiveness of therapy post pathology.

## Introduction

Parkinson’s disease (PD), a highly concerning neurodegenerative disorder affects seven million people worldwide including one million in US alone [1]. Motor symptoms such as tremor, slowness of movements, rigidity, postural instability, and gait impairment are commonly observed in PD patients [2]. Such altered dynamics of gait pattern in PD patients compared to their control counterpart can potentially be exploited to diagnose and quantify longitudinal disease progression.

Gait pattern and characterstics are commonly characterized into three parameter types: 1) spatiotemporal, 2) kinematic, and 3) kinetic [3]. Spatial parameters include stride length, which measures the distance between successive points of heel contact. Cadence, duration of swing, and stance phase are examples of temporal parameters, which provide information regarding abnormality or slowness in completing a gait cycle. Kinematic parameters describe the motion of objects with no consideration to the source of the motion. For example, ankle, knee, and hip angles at heel strike and toe off are kinematic parameters. Kinetic parameters, such as ground reaction force during walking, measure the force that causes the motion.

Two types of sensors have been used in previous studies to analyze gait parameters, first, to directly measure an event within a gait cycle; for example, foot-switch and force sensitive insoles [4], second, to reconstruct the timing of different phases of a gait cycle; for example, gyroscopes and accelerometers applied to the foot [5–6]. In this study, force sensitive insoles were used to measure Vertical Ground Reaction Force (VGRF) data from PD patients and controls during walking. Previous studies have shown alterations in VGRF characteristics while walking in different clinical as well as condition requiring physical activity [7–25]. Besides investigating the gait pattern of PD patients, VGRF has also been used frequently to study the gait of amyotrophic lateral sclerosis (ALS) [7], Huntington’s disease [8], and stroke patients [10] and elderly people [19]. Table 1 summarizes previous studies based on VGRF data as a primary signal along with their area of application and research methods.

**Table 1:** Related work of Gait Analysis using VGRF.

Related Work of Gait Analysis Using VGRF Application Area	Features Used	Method Used
ALS disease [7]	Stride-stride fluctuality	Statistical Analysis (Kruskal-Wallis)
Huntington’s disease [8]	Alpha, computed from DFA	Detrended fluctuation analysis
Bilateral coordination of gait [9]	Gait asymmetry, phase coordination index	Statistical Analysis (General linear models)
PD, Huntington’s, ALS [10]	Coherence, Entropy	Predictive analysis
Running performance [11]	Vertical loading rate, impact/passive peak, active peak	Statistical analysis
Concussion [12]	Peak VGRF	Statistical analysis
Foot ulcers [13]	Total vertical ground reaction force	Statistical analysis
Soccer players [14]	Peak force at foot flat, peak force at toe off, time between hell contact and foot flat, time until toe off	Statistical analysis
Obese [15]	Peak VGRF, VGRF loading rate	Statistical analysis
Sclerosis [16]	VGRF symmetry index	Statistical analysis
Hip arthroplasty [17]	Principal component	Statistical analysis
Hemiplegic patients [18] Young and elderly gait recognition [19]	Peak force at foot flat, peak force at toe off, time between hell contact and foot flat, time until toe off Basic, Kinetic and kinematic	Statistical analysis Predictive analysis
Normal overground and treadmill walking [20]	GRF maxima	Statistical Analysis
Stroke patients [21]	Swing time variability, stride time variability	Statistical Analysis
Heap Arthroplasty patients [22]	Total and average VGRF	Statistical and objective analysis
Lower limb fractures [23]	Principal component	Principal component analysis
PD [24]	Basic, kinetic and kinematic	Predictive analysis
PD [25]	Average gait speed, average stride time, stride time variability, average swing time, average stride length	Predictive analysis

ALS, amyotrophic lateral sclerosis; PD, Parkinson Disease.

Multiple methods have been used to differentiate normal and PD gait patterns using VGRF data. A mathematical model of VGRF time series of PD gait based on an autoregressive model has been proposed by Alkhatib *et al*. [4]. However, this model utilizes information from one sensor, discarding potentially useful information from other force sensors integrated into the shoe. Gait variability of PD patients based on VGRF has been investigated in other studies [25–29], in which a significantly increased variability in duration of gait tasks, such as swing time and stride time, has been observed in PD patients. This work accounts for such variability by including the swing time and stride time variability obtained from the VGRF data.

Machine learning techniques have been employed for classification of normal and PD gait. In Manp’s research [24], an artificial neural network (ANN) with one hidden and one output layer was used to detect PD gait patterns. Basic, kinetic, and kinematic features were fed to the ANN classifier for binary classification of normal and PD patient walking gait. Although they demonstrated good accuracy, their experiment relied on an expensive camera setup, making it cost prohibitive.

The research by Zhang and colleagues [28] conducted machine learning analysis on datasets consisting of VGRF recordings along with SVM and sparse representation-based classifiers to correctly classify PD and healthy gait patterns. Change of VGRF with time beneath heels and toes were extracted as features, ignoring VGRF from other areas of the foot. Classification accuracies of 83.44% and 81.53% were achieved using sparse representation and SVM classifiers, respectively. However, use of additional features considering VGRF characteristics from the whole area beneath the foot is expected to improve the classification performance. Classification of normal and abnormal gait using the k-Nearest Neighbor (kNN) classifier was conducted by Alkhatib *et al*. [29] on the same datasets used by Zhang [28]. In this research, VGRF data from one sensor, instead of an array of sensors, was used to calculate features, and a classification accuracy of 83% was achieved.

This work addresses the shortcomings of previous work by including new features extracted from VGRF sensors, objective feature selection, and determining the performance of different classification methods. We analyzed VGRF time series signal of gait data from an array of sensors on the left foot for both normal and PD patients during walking at normal pace. Distinguishing features were extracted using a feature selection algorithm to achieve the best classifier performance. Four different machine learning classifiers were used to classify the gait pattern between normal and PD patients.

In the remainder of the paper, the experimental method and details of study data are explained in the methodology, followed by the results section. Furthermore, we discuss the merits of our results along with future improvements in the discussion and conclusion sections.

## Methodology

### Database description

The data was recorded by Yogev et al. [30] and was downloaded for analysis from Physionet website [31, 32]. Time series of the VGRF signal recorded from 29 PD patients (Hoehn and Yahr score = 2.3±0.40, UPDRS score = 39.31±12.38 mean age 71.1±8.05 years) and 18 age-matched controls (mean age 71.6±6.6 years) during normal level ground walking were used [31].

**Fig 1:**
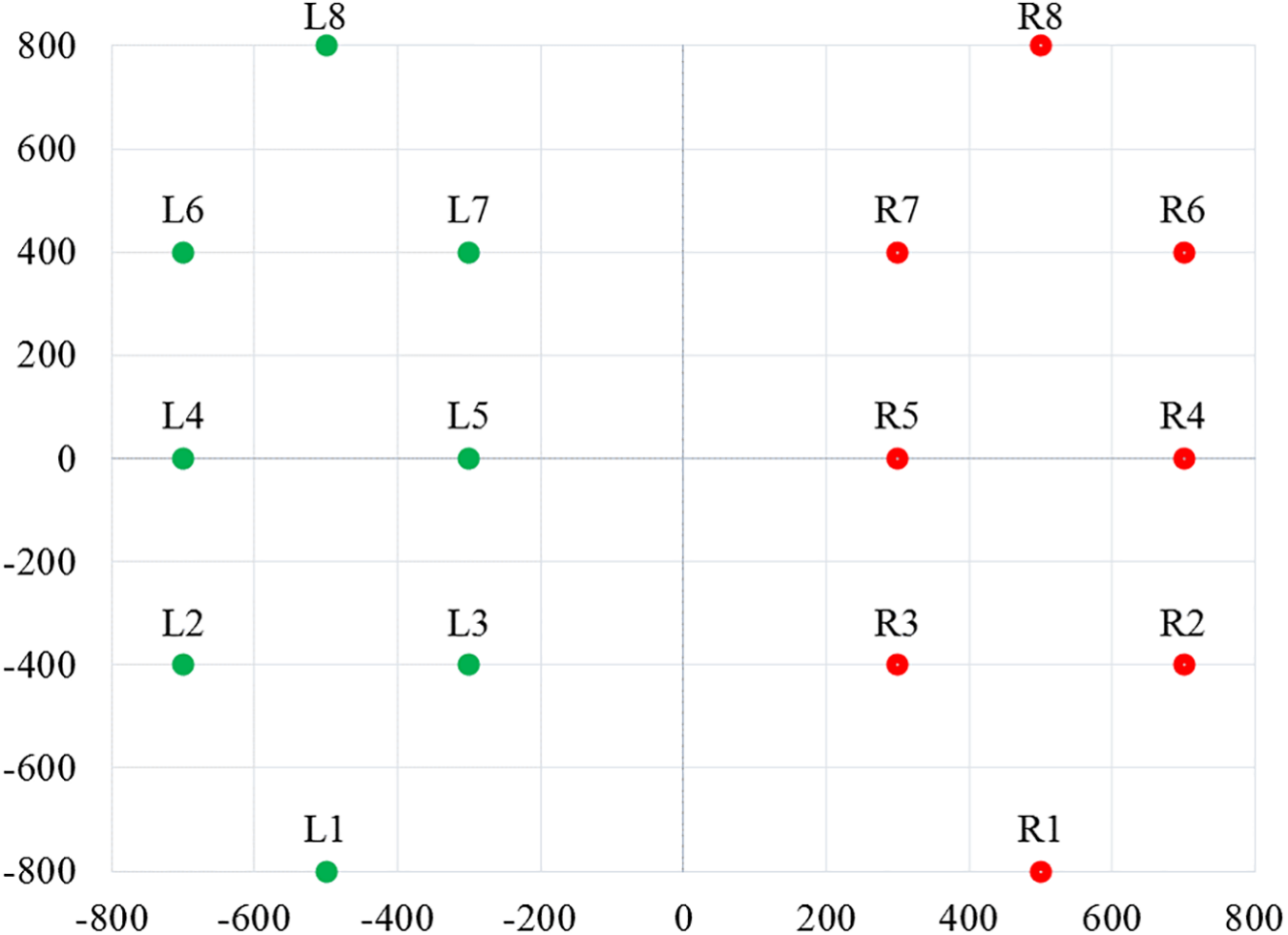
Sensor locations of insoles on the right and left insoles. X- and Y-axes reflect an arbitrary coordinate system to scale the positions of the sensors within each insole.

All the subjects were taking antiparkinsonian medications and their prescriptions were unaltered at the time of experiment. Written consent was collected from all participants, and the study was approved by the Tel-Aviv Sourasky Medical Center Human Studies Committee. As per experimental protocol, the subjects walked at their natural pace on level ground for two minutes, and data was acquired with a sampling frequency of 100 Hz. The system used to collect gait data consisted of eight sensors beneath each foot and a recording unit. A small and light (19 x 14 x 4.5 cm; 1.5 kg) recording unit was carried at the waist. A memory card contained in the unit stored the measurement data during the test, which was later transferred to a computer for analysis. To accurately describe the sensor location inside the insole, it was assumed that the subject was in a comfortable standing position with both legs parallel to each other. Then, coordinates of the sensor location can be displayed as shown in Fig 1. It was assumed that (0, 0) is the origin and lies just between the legs and the person was facing towards the positive side of the Y-axis.

### Data preprocessing and gait cycle segmentation

**Fig 2:**
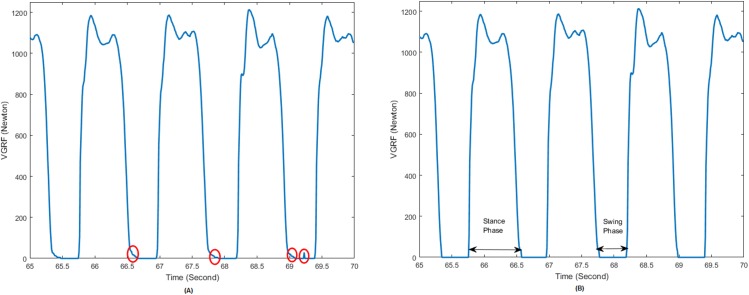
VGRF Signal During Walking. (A) Unfiltered VGRF data. Unwanted VGRF data is circled in red. Right circle represent small amount of VGRF noise value between two stance phases. VGRF noise can also be seen at the end and beginning of stance phase (middle red circle). (B) Filtered VGRF data.

The gait data were processed to remove any extraneous noise or spurious signal. Usually, VGRF values less than 20N are generated from noise; thus, even when the leg is in the swing phase and not exerting any force, there may be some small sensor readout. Therefore, the VGRF time series were filtered such that the VGRF data less than 20N were set to zero in order to reduce the noise and make it easier to segment the gait cycle [33]. As shown in Fig 2, there were several noise signals as circled in red, which were removed after the filtering step.

For gait cycle segmentation, to eliminate the effect of gait initiation, the first 20 seconds of VGRF data was discarded. Then, the time series was divided into individual stride cycles. A stride cycle is the period of time during which a foot touches the ground, goes off the ground, and again makes contact with the ground. It was calculated by taking a sequence of non-zero total VGRF values from all eight sensors followed by a sequence of zero VGRF values in all the sensors. Each gait cycle was further divided into stance phases and swing phases to simplify feature extraction from a particular phase. The foot remains touching the ground during the stance phase; then the foot swings through the air without touching the ground in the swing phase, thus completing a gait cycle.

### Feature extraction

As evident from previous research [21], [27] stride time variability and swing time variability are important parameters in distinguishing a PD patient’s gait from normal gait. Usually, an increased variability of stride time and swing time is observed in PD patients compared to control subjects. The stride time and swing time of each gait cycle were calculated to capture this variability. The time of the combined VGRF value was tracked to separate gait signal into multiple stride cycles. When the combined filtered VGRF value goes to zero after a non-zero value, new gait cycle begins. Therefore, each gait cycle consists of a sequence of zero values (swing phase) followed by a sequence of non-zero values (stance phase).

The coefficient of variation was calculated using the following formula:

CV=(a/b)*100 (1)

where *a* is the mean of a feature calculated from VGRF values among all eight sensors of either the left or right foot and *b* is the standard deviation of the feature.

As PD patients tend to put less pressure during placing the heel strike and toe off than control subjects [29], maximum VGRF at heel strike and toe off for each gait cycle was computed. The mean and standard deviation of the VGRF overall gait cycles were taken as features for classification.

Healthy individuals present a characteristic weight distribution where the center of pressure (CoP) shifts from heel to toe over the course of a stance phase. As PD patients tend to be more flat footed, a transition in CoP shows variation from normal CoP. For this reason, mean and standard deviation of *x*- and y-coordinates of CoP were computed and extracted as features for machine learning classifier. Mean skew and kurtosis values of VGRF gait cycle were also computed. Another feature was the mean peak power of VGRF signal over all the gait cycles in the frequency domain. All features used are listed in Table 2.

**Table 2:** List of features extracted from the vertical ground reaction force data.

Features	Description
Coefficient of Variation (CV) of swing time	*CV*_*swing*_ = (*mean*/*standard deviation*) * 100
Coefficient of Variation (CV) of stride time	*CV*_*stride*_ = (*mean*/*standard deviation*) * 100
Mean Center of Pressure (CoP) of *x*-coordinate (Newton)	$Mean\;of\;{COP}_{x}=\frac{\sum_{i}^{n}s_{ix}*f(s_{ix})}{\sum_{i}^{n}f(s_{ix})}$ where *n* is the number of sensors, *s*_*ix*_ is *x*-coordinate of that sensor, and *f*(*s*_*ix*_) is the VGRF value of sensor i in N
Standard deviation of Center of Pressure (CoP) of *x*-coordinate (Newton)	$Standard\;Deviation\;of\;{COP}_{x}=\frac{\sum_{i}^{n}s_{ix}*f(s_{ix})}{\sum_{i}^{n}f(s_{ix})}$ where *n* is the number of sensors, *s*_*ix*_ is *x*-coordinate of that sensor, and *f*(*s*_*ix*_) is the VGRF value of sensor i in N
Mean Center of Pressure (CoP) of *y*-coordinate (Newton)	$Mean\;of\;{COP}_{y}=\frac{\sum_{i}^{n}s_{iy}*f(s_{iy})}{\sum_{i}^{n}f(s_{iy})}$ where *n* is the number of sensors, *s*_*iy*_ is *y*-coordinate of that sensor and *f*(*s*_*iy*_) is the VGRF value of sensor i in N
Standard deviation of Center of Pressure (CoP) of *y*-coordinate (Newton)	$Standard\;Deviation\;of\;{COP}_{y}=\frac{\sum_{i}^{n}s_{iy}*f(s_{iy})}{\sum_{i}^{n}f(s_{iy})}$ where *n* is the number of sensors, *s*_*jy*_ is *y*-coordinate of that sensor, and *f*(*s*_*iy*_) is the VGRF value of sensor i in N
Mean peak force at heel strike (Newton)	Mean of maximum value of VGRF force of sensors beneath heel for first five sample points in stance phase
Mean peak force at toe strike (Newton)	Mean of maximum value of VGRF force of sensors beneath toe for last five sample points in stance phase
Standard deviation of peak forces at heel strike (Newton)	STD of maximum value of VGRF force of sensors beneath heel for first five sample points in stance phase
Standard deviation of peak forces at toe strike (Newton)	STD of maximum value of VGRF force of sensors beneath heel for first five sample points in stance phase
Mean kurtosis (Second)	Mean kurtosis of the gait cycle duration
Mean skewness (Second)	Mean skewness of the gait cycle duration
Mean Peak power of VGRF signal (Decibel)	Mean maximum power from Power Spectral Density analysis of a VGRF gait cycle

### Feature selection

#### Sequential forward selection

A sequential forward selection algorithm was applied to select an optimal subset of features that provide the best accuracy to detect PD gait. Stride time variability was first added to the empty feature set. For this feature, subsequent cross-validation accuracy to classify PD and control gait was determined. All the other features were added sequentially to the feature vector, and performance of the model was evaluated. If adding extra features resulted in a decrease of classification accuracy, then those features were omitted from final analysis. For optimal feature selection, SVM with linear kernel was selected to predict the model accuracy.

#### Minimum redundancy maximum relevancy feature selection (MRMR)

The minimal-redundancy-maximal-relevance (MRMR) method proposed by Peng *et al*. was used for optimum selection of features for classification [34]. Unlike other top ranking feature selection methods, this method considers relationships among features. Here feature sets, which satisfy maximum relevancy criteria by analyzing mutual information between the features, are selected. Simultaneously, a minimal redundancy condition is added to selected feature sets that are mutually exclusive.

#### Mutual information based feature ranking method

The mutual information based feature ranking algorithm described in Pohjalainen’s work [35] was also tested in this study for feature selection. The algorithm assigns weight to each feature, thus forming a ranking of more relevant feature subsets.

### Classification

In order to differentiate PD gait patterns from normal gait patterns, different machine learning classification techniques were tested to ensure the best accuracy. As a result, we evaluated classification methods, including supervised learning with support vector machines (SVM), an instance-based learning technique called k-nearest neighbor (kNN), random forest, and decision trees to classify the gait patterns of normal and PD patients. Accuracy, sensitivity, specificity, and area under the curve of receiving operator characteristics (ROC) were compared for each classifier.

#### Support vector machine

First, we have applied a state-of-the-art SVM-based classifier. In binary classification, SVM creates a hyperplane that separates data from two different classes. The largest possible distance is established between the separating hyperplane by maximizing the margin, thus creating the separation [36].

The choice of kernel determines the separation boundary of the classes. Radial Basis Function (RBF) or Gaussian kernel are popular algorithms to use as default kernels for any non-linear model. RBF is defined as [37]:

$$K(x,x^{\prime })=exp(-\gamma {\left|\left|x-x\prime \right|\right|}^{2}$$ (2)

where *x* and *x*′ are two training samples of the feature space and γ determines the influence of the squared Euclidian distance between the feature vectors *x* and *x’* to build the hyperplane [37]. Quadratic and cubic kernels are polynomial kernels with degrees of 2 and 3, respectively. Polynomial kernels are defined by [38] as follows:

$$K(x,y)={\left(x.y+1\right)}^{d}$$ (3)

where *x* and *y* are vectors in the input space (*i*.*e*., vectors of features computed from training or test samples) and *d* is the degree of the polynomial. It is generally not advised to consider higher order polynomials because they tend to over-fit the data.

#### K-Nearest neighbour

The next classification technique applied was an instance-based statistical method, kNN. This method is based on the principle that the instances of a dataset will remain in close proximity with the other instances that have similar properties [39]. In this method, a test example is classified by observing the class label of its adjacent neighbors. The kNN find outs the *k* nearest instances to the query instance and identifies its class by finding the single most common class label [40].

#### Decision tree

Decision trees, a hierarchical classifier method, is the simplest and most widely used logic-based classification technique [41]. In this approach, the test data is classified by sorting as trees based on their feature values. The node of the decision tree is the feature of the test data to be classified, and the branches represent a value that the node can predict. Various efficient algorithms have been developed to construct a reasonably accurate decision tree such as Hunt’s algorithm [42], Gini’s diversity index method [43], and relief algorithm [44].

#### Random forest

A random forest [45] is composed of a large number of decision trees which are mainly used to correct the overfitting problem of decision trees. In this technique, multiple decision trees, trained from different subsets of the same training set, are averaged, and overfitting is avoided by reducing the variance of the system, which eventually increases the performance of the final model. The training algorithm works by applying bootstrap aggregating, or bagging techniques, to tree learners.

### Classification experiment

The accuracy of a classification technique is judged based on prediction accuracy. There are three standard methods to evaluate prediction accuracy. The first technique is to split a certain ratio (generally 2/3) of the whole dataset to use as a training set to train the classifier while using the rest of the data as a test set to evaluate the prediction performance. Another technique, cross-validation, divides the dataset into equal-sized mutually exclusive subsets; for one subset, the classifier is trained by the union of other subsets. The average error rate is then used as the error rate of the classifier. The third technique is the leave one out method, which is mainly used for small datasets. This is a particular form of cross-validation where only one instance is used as the test set while all other data are used for training the classifier. For our system, we used the leave one out method to predict the accuracy.

SVM employs kernels to map the data into a higher-dimensional feature space where data can be separated by a hyperplane [46]. As swing time variability and stride time variability are two of the essential features, the model was build first with training the classifier with two features. Additional features were added individually, and the accuracy of the model was tested. When adding a new feature decreased the model performance, it was removed for best model prediction accuracy.

Next, the model was trained with different SVM kernels. In addition to a linear kernel, Gaussian, quadratic and cubic kernels were used to predict the model accuracy with leave one out validation. For kNN, different values for k were tested; the system operated best when the number of neighbors was ten (k = 10). The distance matrix calculation approach was Euclidean, and the distance weight was kept equal. For decision tree, the maximum number of splits was twenty for best performance. The split criterion used were Gini’s diversity index method. Similarly, for random forest technique, for best performance the ensemble method chosen for the proposed system was AdaBoost with the maximum number of splits selected as twenty. The number of learners set at thirty with a learning rate of 0.1.

After selecting the best model for kNN, decision trees, and random forest, all the classification models were trained with the chosen features, and leave one out validation was done on the training model to calculate the prediction accuracy. We also analyzed the system performance by applying dimensionality reduction based feature selection using Principle Component Analysis (PCA). PCA mainly used for retaining most significant features with highest between group variance [47]. Principle components which account for 95% of the variance in the feature matrix were retained during the calculation.

## Results

From the feature selection algorithm, a subset of ten features was selected out of a total of 13 computed features. On applying the sequential forward feature selection algorithm, skewness, kurtosis, and peak power of VGRF features were excluded as adding these features did not improve the classifier performance. On the other hand, MRMR and mutual information based feature ranking methods excluded mean COP of *y*-coordinate, standard deviation of peak force at toe strike and peak power of VGRF. From Table 3, it can be seen that sequential forward selection performs best when these features are tested with SVM classifier with a linear kernel. So features selected with this approach were used for testing with other classifiers

**Table 3:** Comparison of different feature selection methods.

Feature selection method	Selected Features	Accuracy	AUC
Forward Feature Selection	CV swing time, CV stride time, Mean COP_x_, SD COP_x_, Mean COP_y_, SD COP_y_, Mean PF at heal strike, Mean PF at toe strike, SD PF at heal strike, SD PF at toe strike	91.6%	0.94
Minimum Redundancy Maximum Relevancy Feature Selection (MRMR)	CV swing time, CV stride time, Mean COP_x_, SD COP_x_, SD COP_y_, Mean PF at heal strike, Mean PF at toe strike, SD PF at heal strike, Mean kurtosis, Mean skewness	83.1%	0.86
Mutual information based feature ranking method	CV swing time, CV stride time, Mean COP_x_, SD COP_x_, SD COP_y_, Mean PF at heal strike, Mean PF at toe strike, SD PF at heal strike, Mean kurtosis, Mean skewness	83.1%	0.86

CV, Coefficient of Variation; COP_x_, Center of Pressure (CoP) of x-coordinate; COP_y_, Center of Pressure (CoP) of y-coordinate; SD, Standard Deviation; PF, Peak Force.

The results regarding the comparison of different classifiers are listed in Table 4. The metrics used in the study to evaluate the classifier performance are leave-one-out cross-validation accuracy, sensitivity, specificity, and area under the curve (AUC) of ROC.

**Table 4:** Comparison of different classifiers.

Classifier	Accuracy	Sensitivity	Specificity	AUC
SVM (Linear)	91.6%	93.1%	90.1%	0.94
Random forest	89.4%	88.9%	89.7%	0.89
kNN	85.1%	83.3%	86.2%	0.85
Decision tree	87.21%	88.9%	86.2%	0.88

Within SVM, results of evaluation metrics with respect to different kernels are shown in Table 5. The accuracy of PCA-based feature selection on the best performing kernel is also reported in Table 5.

**Table 5:** Comparison of different kernels.

Kernel	Accuracy	Sensitivity	Specificity	AUC
Linear	91.6%	93.1%	90.1%	0.944
Gaussian	91.5%	88.9%	93.1%	0.973
Quadratic	89.4%	88.9%	89.7%	0.952
Cubic	95.7%	94.4%	96.6%	0.980
Cubic with PCA features	93.6%	88.9%	96.6%	0.973

Table 6 illustrates the comparison of the best performing classifier to other two studies that reported classification results on the same dataset. Since sensitivity, specificity, or AUC was not reported in these papers, the classification performance comparison was only based on classification accuracy. As shown in Table 6, the higher classification accuracy between the two other studies is 83.44%, *i*.*e*., about 12% less than the results obtained by our proposed method.

**Table 6:** Comparison with other works on the same database.

Research	Classifier Used	Classification Accuracy
Zhang [19]	LC-KSVD	83.44%
Alkhatib [20]	KNN	83.00%
Proposed method	SVM	95.70%

## Discussion

The goal of this study was to develop an algorithm for VGRF-based gait measurement to distinguish between gait from PD patients and healthy individuals. In contrast with previous studies, the proposed study tested a combination of different classifiers and features to find the optimal set to classify gait of normal and PD patients accurately. Features related to the characteristics of normal and PD gait were obtained by an in-depth study of a PD patient’s foot strike pattern. Features used in previous studies were also taken into account. The classification accuracy was used to assess differences in gait patterns between healthy and PD patients to demonstrate the potential of the extracted features.

As illustrated in Table 4, among various classification techniques, the SVM classifier can differentiate PD from normal gait with the highest accuracy. SVM with linear kernel shows about 2% better performance than the second best classifier, kNN. Several kernels were evaluated to optimize the performance further. Performance metrics of SVM classifier in response to different kernels are shown in Table 5. It is seen from Table 5 that the polynomial kernels performed better than the more popular Gaussian kernel. Among polynomial kernels, cubic kernel shows the best accuracy at 95.7% on training model with 94.4% sensitivity, 96.6% specificity, and an AUC of 0.980. After applying PCA on selected features, subset accuracy dropped about 2.1%. Since the number of features in feature subset is only 10, further dimensionality reduction using principal component analysis results in decreased classification accuracy. Since the accuracy was reduced, we would suggest running the algorithm without PCA. As the dataset was not large enough, instead of dividing it into the train and test sets, leave one out cross-validation accuracy was performed on the feature vector.

Compared to Manap’s results [24], our analysis achieved a similar classification rate (approx. 95%). However, in their study, basic, kinetic, and kinematic parameters of gait were considered, which led to significant increases in cost and computational complexity. When only VGRF data have been taken into account in their study, classification accuracy was reduced to 81%. On the other hand, we obtained 95% accuracy while extracting information from the VGRF time series only.

Table 6 shows that our method outperforms two other studies on the same dataset on each of the evaluation metrics. The better performance of our algorithm is due to the unique selection of features and classifier. None of the other studies considered CoP of PD patient during walking as a discriminative feature to classify PD gait. Also, maximum force during foot placement at the ground and off the ground was not considered in those studies. Our study demonstrated that CoP during the stance phase in combination with gait variability and maximum VGRF feature achieved good classification accuracy. Alkhatib *et al*. achieved 83% classification accuracy by proposing ANN for classifying normal and pathological gait, ignoring SVM [29]. Zhang *et al*. investigated sparse representation based classification algorithm LC-KSVD and SVM [24], [28] and achieved an 83.3% and 81% classification accuracy, respectively, from features extracted from force data of heel and toe from the left and right foot (LRHT). On the other hand, a range of machine learning classifiers with different kernel functions was applied to classify the normal and PD VGRF time series. Also, a large set of features were computed from the dataset, and a subset of useful features was selected to feed into the classifier, which resulted in significantly improved classification accuracy in our study.

It is worth noting that the present study is limited in the disproportionate sample size of control and pathological groups. Furthermore, only vertical component of ground reaction force (GRF) was investigated in our study as the system did not capture GRF in other directions. However, it is evident from our work that vertical component of GRF alone is able to separate the gait pattern of PD and control with reasonable accuracy with the advantage of lower computational complexity.

Our study demonstrated that VGRF time series along with an SVM classifier could lead to accurate prediction of PD gait. Sensors used in the study can be easily integrated with shoes so that the system is very unobtrusive, which would facilitate PD progression monitoring on a daily basis. The pressure insoles which record VGRF signals are low cost, reducing the overall cost of the system.

## Conclusion

In this study, an extensive machine learning approach was investigated on a publicly available dataset of gait data of PD patients and control subjects. By using VGRF, this work proposed a set of meaningful features which can successfully differentiate healthy and pathological gaits. The most suitable classifier was found by testing SVM, random forest, kNN, and decision tree. The best classification performance was obtained from features based on stride and swing phase variability, maximum force at heel strike and toe off, and location of the center of pressure during walking. The results demonstrated by the classification accuracy showed the effectiveness of the proposed approach. Overall, it is believed that the proposed VGRF features-based machine learning approach has the potential for application in clinical diagnosis and longitudinal monitoring.

It is already proved in Ota et al.’s study [48] that non-linear properties like fractal properties of PD gait contain significant information. In our future work, non-linear properties of PD gait will be analyzed, and fusion of non-linear and linear features will be investigated to see whether this leads to improvement in classification accuracy for PD diagnosis.
